# Plants with Antimicrobial Activity against *Escherichia coli*, a Meta-Analysis for Green Veterinary Pharmacology Applications

**DOI:** 10.3390/microorganisms12091784

**Published:** 2024-08-28

**Authors:** Rosario De Fazio, Francesca Oppedisano, Giulia Caioni, Bruno Tilocca, Cristian Piras, Domenico Britti

**Affiliations:** 1 Department of Health Sciences, “Magna Græcia University” of Catanzaro, Campus Universitario “Salvatore Venuta” Viale Europa, 88100 Catanzaro, Italy; rosario.defazio@studenti.unicz.it (R.D.F.); foppedisano@unicz.it (F.O.); tilocca@unicz.it (B.T.); britti@unicz.it (D.B.); 2 Department of Bioscience and Technology for Food, Agriculture and Environment, University of Teramo, 64100 Teramo, Italy; gcaioni@unite.it; 3 CISVetSUA, “Magna Græcia University” of Catanzaro, Campus Universitario “Salvatore Venuta” Viale Europa, 88100 Catanzaro, Italy

**Keywords:** *E. coli*, antimicrobial plants, antibiotic resistance, green veterinary pharmacology

## Abstract

Antimicrobial resistance (AMR) poses a critical global health threat, necessitating innovative strategies to combat infectious diseases. Plants and their extracts offer alternatives/support to traditional antibiotics, and their diverse phytocomplexes with antimicrobial properties can be exploited. The Mediterranean hosts a high number of biodiverse plant species known for their bioactive compounds. This study focuses on identifying plant species and phytochemical constituents with antimicrobial activity against *Escherichia coli* (*E. coli*). Utilizing a systematic literature review and meta-analysis approach, we screened 3037 entries, yielding 70 studies suitable for MIC (minimum inhibitory concentration) annotation. The results highlighted *Lavandula* spp., *Plectranthus* spp. and *Lupinus jaimehintoniana* as the most effective plants with average MICs of, respectively, 0.144 mg/mL, 0.260 mg/mL, and 0.140 mg/mL. These results might help to fight AMR via the discovery of complementary natural antimicrobial agents to support the development of green veterinary pharmacology. Further exploration of these resources promises valuable insights for future support to antimicrobial strategies.

## 1. Introduction

The world now faces a serious threat from antimicrobial resistance, which makes traditional antibiotics less effective and limits our ability to fight infectious diseases.

Antimicrobial resistance (AMR) has significantly increased morbidity and mortality in both humans and animals, posing serious challenges for future treatments of infection and affecting animal health and productivity. The use of antimicrobials in animals is considered a major factor in the emergence of AMR worldwide, with many high-income countries reporting extensive antimicrobial use and resulting resistance in animals. To reduce antibiotic use in animals, it is crucial to address risk factors for infectious diseases, to work on the genetic background of breeds and farm management practices, and to use alternative remedies when possible [1,2,3,4,5,6,7,8].

*E. coli* are Gram negative bacteria that are commonly present the gastrointestinal tracts of humans and animals. In beef cattle, *E. coli* are generally harmless, though certain strains can cause diarrhea in young calves. Similarly, most *E. coli* strains in humans are benign, but some can cause illness. In cattle, the most significant strains are those producing Shiga toxins (STECs), such as *E. coli* O157, with cattle serving as reservoirs for these bacteria [9]. In humans, STEC infections can range from being asymptomatic to causing diarrhea, hemorrhagic colitis (bloody diarrhea), or hemolytic uremic syndrome, which can result in kidney failure. Occasionally, STEC infections can be fatal, particularly in children, the elderly, or immunocompromised individuals [10]. The primary sources of foodborne STEC outbreaks in humans include raw or undercooked ground meat products, raw (unpasteurized) milk, and the fecal contamination of vegetables [11].

The emergence of extended-spectrum β-lactamase (ESBL)-producing Enterobacteriaceae, which can hydrolyze key antimicrobials like cefotaxime, ceftriaxone, ceftazidime, and cefepime, is primarily due to antibiotic pressure in both human and veterinary medicine [12]. The use of third- and fourth-generation cephalosporins was linked to resistance in *E. coli* in humans, while tetracycline and polymyxin resistance in *E. coli* from animals was associated with corresponding antimicrobial use in animals. This resistance is mainly mediated by acquired ESBL genes located on mobile genetic elements and is often linked to resistance genes against multiple antimicrobial families [13].

The need to address this situation has led to several research initiatives for alternative solutions, such as substances derived from plants [14]. For this purpose, a wide panel of different plants are available, and their biodiversity in a certain region is influenced by environmental factors as latitude, altitude, temperature, precipitation, and many others [15,16].

From this perspective, the Mediterranean region’s heterogenous topography has promoted the growth of consistent plant biodiversity. For example, elevation is directly correlated with increases in rainfall and a lower temperature, which can lead to the growth of diverse plant communities. In the alpine zone, for instance, plant diversity is strongly determined by the local topography, microclimate, and various factors such as the direction of slope faces, which is a significant factor in determining species composition [17]. Elevation can also provide protection for mountain-specific vegetation, acting as a refuge from human-induced disturbances [18].

Wide latitude ranges and the presence of mountains and coastal regions are favorable conditions for the development of different types of ecosystems [19]. In these habitats, plants like myrtle (*Myrtus communis*), lavender (*Lavandula* spp.), rosemary (*Rosmarinus officinalis*), and other aromatic herbs flourish [20].

Bioactive substances, such as terpenes, flavonoids, phenolics, and alkaloids, can be found in phyto-complexes of aromantic plants and are necessary as defensive mechanisms against microbial diseases and environmental challenges. These bioactive substances have the ability to interfere simultaneously with virulence factors, target vital microbial functions, and damage cellular structures, all of which lower the likelihood that resistance will emerge [7,14,21,22,23].

Evaluating the effectiveness of these plants or their extracts against microorganisms that compromise animal productivity efficiency may provide a good substitute for traditional antimicrobial treatment methods. It might also help prevent the creation of new antibiotic resistance mechanisms and events. Many plants and plant extracts have been already studied for their anti-microbial properties; however, the literature about these findings is very heterogeneous and difficult to interpret. In this matter, plants active against one of the major Gram-positive pathogens have already been studied. The plant species and compounds with lower minimum inhibitory concentrations (MICs) against *Staphylococcus aureus* were recently investigated through a systematic literature review and meta-analysis yielding good candidates that are currently being tested as possible GVP interventions [7].

However, even if many plants have been often investigated for their bioactivity against Gram-negative pathogens, a comprehensive summary of such information from the literature is still missing. Therefore, the aim of the present work was to screen the published (peer-reviewed) literature about plants tested against *E. coli* in order to identify the plant species and the respective phytochemical constituents that present the lowest MIC values against the growth of these pathogens. The applicability of the obtained results will be necessary to develop alternative or supportive strategies to be used to minimize the causes of antimicrobial resistance.

## 2. Materials and Methods

The list of plants identified by Piras et al. [14] served as a guide for our bibliographic searches. This collection of plants actually serves as the foundation for new, alternative strategies that green veterinary pharmacology applications will employ to combat bacterial illnesses. In 2022, this list of plant extracts that were effective against the most common diseases for animal husbandry and infections was released [14]. The list included both plants present in Italian territory and pathogens including Gram-positive and Gram-negative bacteria. In particular, it was discovered that 20 plants were active against *E. coli*, raising the question of which plants had the lowest MICs. Here, the MIC values that have been previously annotated in the literature were registered using a novel systematic review approach to determine which ones could be used for a GVP strategy. In order to obtain the most comprehensive information possible, the new searches were conducted without time or location restrictions. The new searches were performed using the scientific names of the plants in the following list: *Daucus carota* subsp*. Maximus* [24]; *Cytinus hypocistis* [25]; *Matthiola incana* (L.) *R.Br.* subsp. *incana* (*Brassicaceae*) [26]; *Lavandula × intermedia* [27]; *Laurus nobilis* [28]; *Glycyrrhiza glabra* L. [29]; *Malus domestica* var. *Annurca* [30]; *Teucrium genus* (*Germander*) [31]; *Daucus carota* subsp. *maximus* [24]; *Isatis tinctoria* L. (*Brassicaceae*) [32]; *Garlic* (*Allium sativum* L.) [33]; *Thymus vulgaris* L. [34]; *Plectranthus barbatus and Plectranthus caninus* [35]; *Rapa Catozza Napoletana* (*Brassica rapa* L. *var. rapa DC.*) [36]; *Daphne gnidium* L. [37]; *Calycotome villosa* [38]; *Hyssopos officinalis* L. [39]; *Achillea ligustica* [40]; and *Lupinus* spp. [41] from Piras et al. [14]). “*E. coli*” (e.g., garlic (*Allium sativum* L.) and *E. coli*) was searched for in PubMed, Web of Science, and Scopus. For every search, a separate file containing all of the entries was saved. The files of each plant were uploaded to Rayyan (https://www.rayyan.ai/), which was accessed on 17 July 2023. Following this, all of the files were exported as a single “.ris” file to combine all of the search results into a single file. After that, the acquired file was transferred to Mendeley Desktop (version 1.19.8) for a manual inspection and the elimination of duplicates. After exporting the filtered file, it was uploaded to Rayyan (https://www.rayyan.ai/, accessed on 17 July 2023) to be used to search for keywords and screen pertinent publications for inclusion in the meta-analysis.

“MIC”, “MICs”, and “plant scientific name” (genus/species) were the keywords used for Rayyan searches. Using this strategy, it was possible to identify scientific articles pertaining to each plant that contained the terms “MIC” and “MICs” in the title, keywords, or abstract, and likely involved studies or evaluations of the MIC. Following this selection procedure, MIC detection was manually assessed for each record.

This study included whether the MIC value against *E. coli* was clearly indicated. Two independent reviewers completed this process on their own, while another referee cross-validated the results by selecting 15 randomly selected recorded values. The publication title and the MIC values for every plant, as documented in each separate study, were annotated in an Excel file. The same spreadsheet was used to determine the mean and standard deviation (SD), and OpenMeta[Analyst] (http://www.cebm.brown.edu/openmeta/, 15 January 2024) was used to further analyze the results and create the forest plots.

## 3. Results

All the plants annotated in the previously published review were individually searched for along with *E. coli* in three different databases (see the Methods section). The search produced 3037 entries; reviews and other article types were excluded using Ryyan filtering. A total of 2997 research articles were obtained after filtering. All other duplicates were removed with Mendeley filtering, yielding a final list of 2022 single records whose abstracts were manually screened by two independent reviewers to find the MIC used in each.

The inclusion criteria adopted were as follows: research articles with a clear indication of an experimentally recorded MIC (any method, in vitro) against *E. coli*. We excluded review articles, all articles in which the MIC against *E. coli* was not clearly recorded and reported, and all articles with insufficient methodological details. The publication type (e.g., “Review”) was filtered using the Rayyan exclusion tool. The methodological details and the MICs were manually checked by the referees.

**Figure 1:**
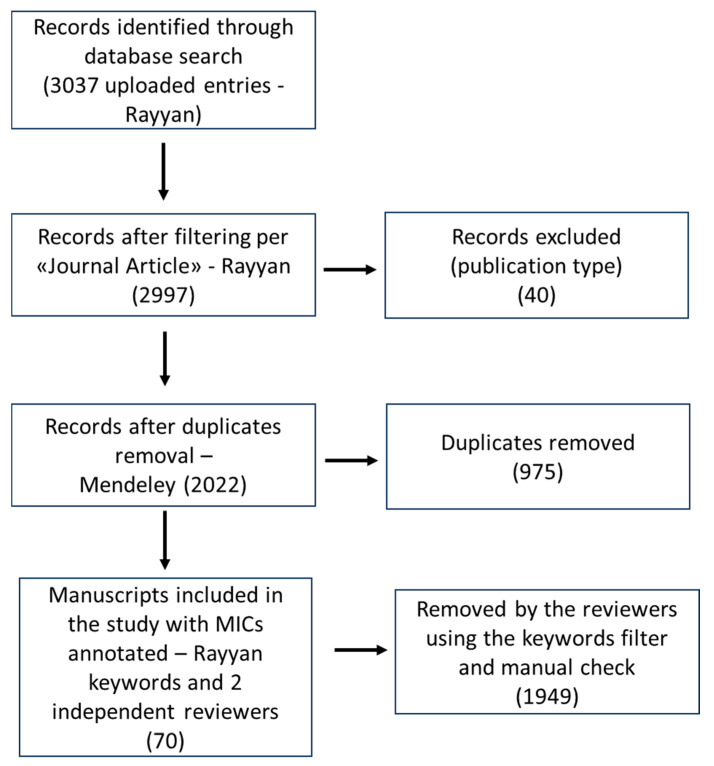
PRISMA diagram showing the records included in the bibliographic research and the filtering steps.

The workflow regarding the inclusion criteria and filtering is shown in the following PRISMA diagram (Figure 1).

The total number of records for each plant with the keywords was detected using the inclusion and exclusion system of the Rayyan tool. The number of total records for each plant is indicated in the second column of Table 1. The third column indicates the number of recorded MIC values manually annotated by each reviewer. The keywords used for the filtering of these records were “MIC”, “MICs”, and “plant name”.

**Figure 2:**
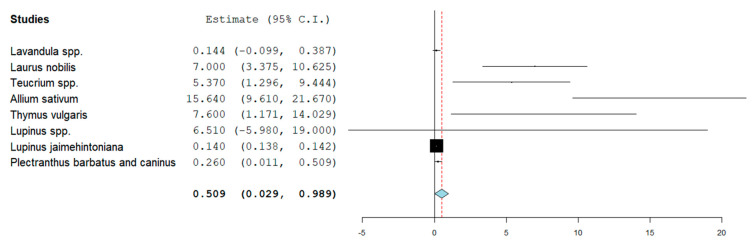
Forest plot showing the representation of the average recorded MIC for each plant extract included in this study. The power (which is related to the number of studies considered) is indicated by the weight (size) of the box.

The MIC values (mg/mL) were manually registered and saved in the Supplementary File S1. For a more comprehensive view, the obtained results are graphically represented in Figure 2 below, which reports the average MIC values for each plant against *E. coli.* Along with the MIC values, the confidence intervals and the power are indicated, which are represented by the black box in each line.

The average values of the annotated MICs yielded the forest plot represented in Figure 2. Among 18 plants genres/species, three showed MICs below 1 mg/mL. Among the 48 records mentioning *Lavandula* spp. as active against *E. coli*, eight were considered relevant to this study, yielding an average MIC of 0.144 mg/mL among tested *E. coli* strains. Eleven total records reported the antibacterial activity of *Plectranthus barbatus* and *Plectranthus caninus*. Even though these plant species are not endemic to the Mediterranean area, they were successfully cultivated in Northern Italy and subsequently investigated to obtain their MICs against *E. coli*. Overall, three recorded MIC values were included, resulting in an average MIC of 0.260 mg/mL. Lupinus is widely studied in the Mediterranean region, and, in total, 53 scientific outputs were included in the study. In order to deconvolute and simplify the results during the previous systematic review [14], we decided to merge all the results to the genus level. In this experimental work, the searches were not restricted to the geographical area and allowed for the detection of *Lupinus jaimehintoniana* (which is native to Mexico), yielding three MIC values with an average of 0.140 mg/mL.

Table 2 below shows a summary of the three most effective plants along with the five most abundant compounds detected in their respective extracts.

## 4. Discussion

This systematic literature review and meta-analysis aimed to identify plant species and phytochemical constituents with the lowest minimum inhibitory concentrations (MICs) against *E. coli*, potentially contributing to green veterinary pharmacology (GVP) as an alternative to traditional antibiotics. Our findings highlight several promising plant species and underscore the rich biodiversity of the Italian peninsula as a valuable resource for combating antimicrobial resistance.

Among a total of 3037 entries, 70 were used for the annotations of the plants’ MICs against *E. coli*.

*Lavandula* spp. (lavender) exhibited antimicrobial activity against *E. coli*, with an average MIC of 0.144 mg/mL. This finding aligns with previous studies that have reported the strong antimicrobial properties of lavender essential oils (EOs), primarily attributed to compounds such as linalool and linalyl acetate. *Plectranthus barbatus* and *Plectranthus caninus* demonstrated significant antimicrobial activity with an average MIC of 0.260 mg/mL. These species, although not endemic to the Mediterranean area, have been successfully cultivated in Northern Italy and show promising results against *E. coli*. This highlights the potential of non-native species adapted to local conditions as sources of bioactive compounds.

*Lupinus* spp. (lupine), widely studied in the Mediterranean region, yielded an average MIC of 6.510 mg/mL. This result is yielded mainly by the annotation of the MICs from the studies that were performed on the extracts from the seeds. Surprisingly, data from *Lupinus jaimehintoniana*, a species native to Mexico, reported the lowest recorded MICs (0.140 mg/mL). This study was based on the alcoholic extraction of lupine leaves rather than seeds and was focused on the enrichment of bioactive alkaloids. The authors found that the MICs of the alkaloids’ enriched parts were lower, but only the dataset related to the alcoholic extraction without further enrichment was reported.

*Lavandula* spp. (lavender) essential oil demonstrates significant antimicrobial activity [44,45,46,47,48,49,50], largely due to its major constituents: linalool (14.93%), camphor (14.11%), linalyl acetate (11.17%), and eucalyptol (10.99%). Each of these compounds contributes uniquely to the oil’s overall efficacy against a broad spectrum of microbial pathogens [51]. Linalool, a monoterpene alcohol, is known for its ability to disrupt microbial cell membranes by integrating into the lipid bilayer, which increases membrane fluidity and permeability, causing the leakage of vital cellular contents and resulting in cell lysis [52]. Additionally, linalool interferes with microbial metabolic pathways, inhibiting the synthesis of essential molecules necessary for cell function and replication. Its antimicrobial effectiveness is often enhanced when combined with other essential oil constituents, demonstrating synergistic effects that potentiate the overall efficacy of the oil.

Camphor, a bicyclic monoterpene ketone, also plays a crucial role in the antimicrobial activity of lavender essential oil. It increases the permeability of microbial cell membranes, facilitating the entry of other antimicrobial agents, which leads to cell death [53,54,55].

Linalyl acetate, an ester of linalool, contributes to its antimicrobial properties through its hydrophobic nature, allowing it to integrate into and disrupt the lipid bilayers of microbial membranes, compromising their structural integrity. When combined with linalool, linalyl acetate enhances the antimicrobial potency of the essential oil through synergistic interactions, providing a multi-targeted attack on microbial cells [56,57,58].

Eucalyptol, also known as 1,8-cineole, is a cyclic ether and monoterpenoid with significant antimicrobial properties. Eucalyptol penetrates and disrupts microbial cell membranes, leading to increased permeability and the leakage of cellular contents. It also inhibits efflux pumps in bacteria, which are responsible for expelling antimicrobial agents, thus increasing bacterial susceptibility to these agents. Additionally, eucalyptol’s anti-inflammatory properties can enhance the healing process in microbial infections, providing a supportive role in its antimicrobial activity [59,60,61].

The genus *Plectranthus* (Lamiaceae) includes more than 300 mainly spontaneous herbaceous species (i.e., *Plectranthus barbatus*, *Plectranthus caninus*, *Plectranthus amboinicus,* and *Plectranthus laxiflorus*) mainly distributed in tropical and equatorial countries, as described by Gelmini and colleagues. *Plectranthus barbatus* was cultivated in Northern Italy [35].

*Plectranthus barbatus* (*Coleus forskohlii*) and *Plectranthus caninus* (*Coleus canina*) essential oils share several key compounds that contribute to their aromatic and therapeutic properties. Both oils commonly contain α-Pinene and β-Pinene, which have antimicrobial effects [62,63,64,65].

Camphor and 1,8-Cineole (eucalyptol) are other shared components and were previously described in this article, as their presence was also detected in lavender. β-Caryophyllene demonstrated a potent antibacterial effect against all tested bacterial strains, with MIC values ranging from 3 to 14 μM [66,67,68].

Along with these compounds, the antimicrobial activities of Germacrene D and α-Humulene were demonstrated [69,70,71]. The synergy of these compounds can lead to a broader spectrum of antimicrobial activity.

For *Lupinus* spp., we decided to include the entire genus because of the multitude of plants that present bioactivity that have been studied in the Mediterranean region [41,72,73,74]. This decision allowed for the inclusion of *Lupinus jaimehintoniana*, which is an autochthonous plant from North America, which was the most effective plant [41].

The *Lupinus* genus was searched entirely (i.e., including all of the species within it) because it has been widely used and studied in the Mediterranean area [41].

One of the most important features of lupine extracts is linked to their alkaloid content in leaves, seeds, and shoots [43]. In particular, shoots and phloem were reported to have the lowest MICs [43]. Among the alkaloids found, the most concentrated were lupanine, 5,6-dehydrolupanine, d-thermopsine, and sparteine. These alkaloids from lupine species were tested for their antimicrobial activity, confirming the inhibition of the growth of *K. pneumoniae* and *P. aeruginosa* [41].

A more comprehensive representation of the composition of the five most abundant essential oils of these plants is represented in Table 2. As shown in the second column, even if the MIC of each extract was below 1 mg/mL, it is still generally higher than the MICs against conventional antibiotics, unless resistance occurs. Moreover, there is a substantial difference in the development of resistance mechanisms towards a single molecule or against phytocomplexes that have multiple molecular targets.

These indicated MICs are related to the use of each EO. The purpose of this study is to detect the most effective ones that can be used as a blend to improve their action spectrum and, possibly, efficacy against mainly *E. coli* and other Gram-negative pathogens. Potential formulations might be used for the treatment of all types of animal diseases that can be treated with topical formulations caused by Gram-negative pathogens (e.g., otitis externa, pyoderma, or bovine mastitis, when the involvement of a Gram-positive pathogen is excluded). Moreover, as EU legislation (EU-Regulation 2019/6) is incomplete for the regulation of the market authorization of herbal veterinary medical products (https://www.cost.eu/actions/CA22109/ 29 July 2024) and, as the toxicity of the proposed essential oils still needs to be evaluated, we would like to mention that this represents the first step towards future trials on their effectiveness, toxicity, and dose–response assessments.

## 5. Conclusions

In conclusion, the systematic exploration of flora that might be able to grow in Italy and produce phytochemicals with antimicrobial activity has revealed promising candidates against *E. coli*, *Lavandula* spp., *Plectranthus* spp., and *Lupinus* spp. These findings underscore the importance of biodiversity in addressing global health challenges and suggest avenues for further research and development. The bioactive properties of these plants could be used in synergy as sustainable alternatives or parallel co-adjuvants to conventional antibiotics, supporting efforts to combat microbial resistance and enhance public health strategies worldwide.

## Figures and Tables

**Table 1:** Number of publication records and recorded MIC measurements for each plant.

Plant	Total Records	Records Included
*Daucus carota* subsp. *Maximus*	121	1
*Cytinus hypocistis*	1	0
*Matthiola incana*	4	0
*Lavandula* spp.	48	8
*Laurus nobilis*	70	14
*Glycyrrhiza glabra* L.	17	0
*Malus domestica var. Annurca*	2	0
*Teucrium* spp.	67	12
*Isatis tinctoria* L.	3	1
*Allium sativum* L.	381	31
*Thymus vulgaris* L.	66	16
*Plectranthus barbatus* and *Plectranthus caninus*	11	3
*Brassica rapa*	49	1
*Daphne gnidium* L.	3	1
*Calycotome villosa*	2	0
*Hyssopos officinalis* L.	1	0
*Achillea ligustica*	1	0
*Lupinus* spp.	53	3

**Table 2:** MICs and composition of the most effective plant extracts against *E. coli*.

Plant	Average MIC mg/mL	Five Most Abundant Compounds	References
*Lavandula* spp.	0.144	Linalool	Essential Oil of Lavandula officinalis: Chemical Composition and Antibacterial Activities [42]
		Camphor	
		Linalyl acetate	
		Eucalyptol	
		4-Terpinenol	
*Plectranthus* spp.	0.26	1-octen-3-ol	GC–MS characterisation and biological activity of essential oils from different vegetative organs of *Plectranthus barbatus* and *Plectranthus caninus* cultivated in north Italy [35]
		anethol	
		b-caryophyllene	
		terpinen-4-ol	
		D germacrene	
*Lupinus jaimehintoniana*	0.140	Lupanine	Alkaloid Profile, Antibacterial And Allelopathic Activities Of *Lupinus jaimehintoniana* B.L. Turner (Fabaceae). [43]
		5,6-dehydrolupanine	
		d-thermopsine	
		Sparteine	
		Nuttalline	

## Data Availability

The original contributions presented in the study are included in the article/supplementary material, further inquiries can be directed to the corresponding author.

## Supplementary Materials

The following supporting information can be downloaded at: https://www.mdpi.com/article/10.3390/microorganisms12091784/s1.
